# Next-Generation Sequencing and MALDI Mass Spectrometry in the Study of Multiresistant Processed Meat Vancomycin-Resistant Enterococci (VRE)

**DOI:** 10.3390/biology9050089

**Published:** 2020-04-27

**Authors:** Carolina Sabença, Telma de Sousa, Soraia Oliveira, Didier Viala, Laetitia Théron, Christophe Chambon, Michel Hébraud, Racha Beyrouthy, Richard Bonnet, Manuela Caniça, Patrícia Poeta, Gilberto Igrejas

**Affiliations:** 1 Department of Genetics and Biotechnology, University of Trás-os-Montes and Alto Douro (UTAD), 5000-801 Vila Real, Portugal; carolinasabenca@hotmail.com (C.S.); telmaslsousa@hotmail.com (T.d.S.); soraia_oliveira_90@hotmail.com (S.O.); 2 Department of Animal and Veterinary Science, University of Trás-os-Montes and Alto Douro (UTAD), 5000-801 Vila Real, Portugal; ppoeta@utad.pt; 3 Functional Genomics and Proteomics Unit, University of Trás-os-Montes and Alto Douro (UTAD), 5000-801 Vila Real, Portugal; 4 LAQV-REQUIMTE, Faculty of Science and Technology, University Nova of Lisbon, 2829-516 Lisbon, Caparica, Portugal; 5 INRAE, Plateforme d’Exploration du Métabolisme, composante protéomique (PFEMcp), 63122 Saint-Genès Champanelle, France; didier.viala@inrae.fr (D.V.); christophe.chambon@inrae.fr (C.C.); michel.hebraud@inrae.fr (M.H.); 6 INRAE, UR0370 Qualité des Produits Animaux (QuaPA), 63122 Saint-Genès Champanelle, France; laetitia.theron@inrae.fr; 7 INRAE, UMR0454 Microbiologie Environnement Digestif Santé (MEDiS), Université Clermont Auvergne, 63122 Saint-Genès Champanelle, France; 8 Centre National de Référence de la Résistance aux Antibiotiques, Centre Hospitalier Universitaire, 63003 Clermont-Ferrand, France; rbeyrouthy@chu-clermontferrand.fr (R.B.); rbonnet@chu-clermontferrand.fr (R.B.); 9 UMR1071 INSERM, USC1382 INRAE Microbiologie Intestin Inflammation et Susceptibilité de l’Hôte (M2iSH), Université Clermont Auvergne, 63001 Clermont-Ferrand, France; 10 National Reference Laboratory of Antibiotic Resistances and Healthcare Associated Infections, National Institute of Health Dr. Ricardo Jorge, 1649-016 Lisbon, Portugal; Manuela.Canica@insa.min-saude.pt; 11 Centre for the Studies of Animal Science, Institute of Agrarian and Agri-Food Sciences and Technologies, Oporto University, 4051-401 Oporto, Portugal

**Keywords:** *Enterococcus* spp., processed meat, antibiotic resistance, next-generation sequencing, MALDI-TOF MS

## Abstract

Vancomycin-resistant enterococci (VRE), due to their intrinsic resistance to various commonly used antibiotics and their malleable genome, make the treatment of infections caused by these bacteria less effective. The aims of this work were to characterize isolates of *Enterococcus* spp. that originated from processed meat, through phenotypic and genotypic techniques, as well as to detect putative antibiotic resistance biomarkers. The 19 VRE identified had high resistance to teicoplanin (89%), tetracycline (94%), and erythromycin (84%) and a low resistance to kanamycin (11%), gentamicin (11%), and streptomycin (5%). Based on a Next-Generation Sequencing NGS technique, most isolates were *vanA*-positive. The most prevalent resistance genes detected were *erm*(B) and *aac*(6’)-*Ii,* conferring resistance to the classes of macrolides and aminoglycosides, respectively. MALDI-TOF mass spectrometry (MS) analysis detected an exclusive peak of the *Enterococcus* genus at m/z (mass-to-charge-ratio) 4428 ± 3, and a peak at m/z 6048 ± 1 allowed us to distinguish *Enterococcus* *faecium* from the other species. Several statistically significant protein masses associated with resistance were detected, such as peaks at m/z 6358.27 and m/z 13237.3 in ciprofloxacin resistance isolates. These results reinforce the relevance of the combined and complementary NGS and MALDI-TOF MS techniques for bacterial characterization.

## 1. Introduction

Enterococci are commensal bacteria known for their incidence in the gastrointestinal tract of humans and animals [1]. *Enterococcus* spp., more specifically *Enterococcus faecium* and *Enterococcus faecalis*, have stood out as the main nosocomial pathogens [2]. Due to their high adaptive capacity, they can be present in different environments, such as soil and water, and have also been found in food products [3]. Vancomycin-resistant enterococci (VRE) are one of the greatest clinical challenges today, because VRE often possess determinants that confer resistance to several classes of antimicrobials and also because of their malleable genome that allows them to easily acquire antibiotic resistance genes, via plasmids and transposons [2,4,5].

The increasing demands for meat lead to the use of antibiotics as food promoters in livestock, which, in turn, lead to a selective pressure increase in livestock gut microbiota for antibiotic-resistant bacteria [6]. A relationship between the use of antibiotics in farm animals and bacterial resistance has been observed in several studies and clinical trials. Antibiotic-resistant bacteria, from animal sources, have been found in meat products available in food retail stores and as a cause of clinical infections and subclinical colonization in humans [7]. For years, avoparcin, a glycopeptide antibiotic analogue of vancomycin, was used in European countries as a growth promoter in animal production [2]. Despite the banning of avoparcin in 1997 for animal use, it’s intense use probably potentiated the prevalence of VRE in animals. Bacterial resistance has implications for human health, generally affecting agricultural workers, through direct contact and passing through the environment and food products [6].

Infections with VRE are more difficult to treat than infection with vancomycin-susceptible enterococci (VSE) [8,9]. The resistance to vancomycin can be mediated by nine different types of van cluster genes (*vanA* -*B*, -*C*, -*D*, -*E*, -*G*, -*L*, -*M,* and -*N*) and we can distinguish them based on their physical location (encoded in the core genome or on a mobile genetic element); the level of resistance they confer; the specific glycopeptides to which they confer resistance (often distinguished operationally as providing resistance to both vancomycin and teicoplanin, or providing resistance to vancomycin but not teicoplanin); whether resistance is inducible or constitutively expressed; and the type of peptidoglycan precursor that is produced by their gene products. [10,11]. While the *vanC* gene characterizes the natural intrinsic resistance in some enterococci species, the others encode acquired resistance [12].

As mentioned, enterococci are intrinsically resistant to aminoglycosides and can acquire resistance genes to other antimicrobial classes [13,14]. However, high-level resistance to aminoglycosides (HLGR) is usually acquired on a mobile element that encodes an aminoglycoside-modifying enzyme. Such an enzyme is usually the bifunctional aminoglycoside-modifying enzyme, AAC(6′)-Ie-APH(2′′)-Ia, which is encoded by the *aac*(6′)-*Ie*-*aph*(2′′)-*Ia* gene, reducing the effect of aminoglycosides except for streptomycin [9,11]. Furthermore, among gentamicin-resistant strains, three aminoglycoside resistance genes, i.e., *aph*(2′′)-*Ib*, *aph*(2′′)-*Ic*, and *aph*(2′′)-*Id*, have been reported. The *ant*(4′)-*Ia* and *aph*(3′)-*IIIa* genes encode resistance to various aminoglycosides as well [4,9,15]. Thus, in spite the intrinsic resistance, aminoglycosides are still used in the treatment of enterococcal infections but at high concentrations to prevent intrinsic resistance. 

Moreover, enterococci also show resistance to macrolides, and their major determinants of resistance are ribosomal target modification by 23S rRNA methylases encoded by *erm* genes and efflux pump systems encoded by *msr* and *mefA/E* genes [9,16]. Enterococci that carry erm genes can result in inducible or constitutive resistance to all macrolide, lincosamide, and streptogramin B antibiotics. Alternatively, resistance to streptogramin B and some macrolide antibiotics is induced by the *msrA* gene [9,17].

Antibiotic resistance has been explored in many microorganisms as has the study of proteins associated with different mechanisms [18,19]. MALDI-TOF mass spectrometry (MS) produces spectra capable of discriminating strains of various types of Gram-positive and Gram-negative bacteria, such as methicillin-resistant *Staphylococcus aureus* (MRSA) [20] and vancomycin-resistant enterococci, respectively [21], demonstrating great utility for the subtyping of strains [22].

As antibiotic-resistant bacteria can make it difficult to treat patients with complex infections, rapid and effective detection of resistance mechanisms can be vital in choosing the best antimicrobial [19]. For this reason, the proteomics approach becomes an extremely important tool for the identification of expressed proteins in a differentiated way and for the discovery of new biomarkers in order to understand which proteins are more or less abundant in a diseased state compared to the healthy state [23,24]. As biomarkers are discovered, inclusive information on the nature of proteins and their expression in relation to antimicrobial agents and resistance mechanisms should be stored in databases [25].

Thus, we aimed to identify VRE from processed meat (hamburgers, meatballs, and minced meat), investigate antimicrobial resistance, and find biomarkers of resistance in the *Enterococcus* genus, which will also be important for VRE from other reservoirs, as bacteria that can be studied in a One Health context.

## 2. Materials and Methods

### 2.1. Samples and Bacterial Isolation

Between January 2018 and October 2019, one hundred and twenty-nine packages of different processed meat samples were purchased from six different supermarkets in northern Portugal. We analyzed a total of 43 packs of each processed meat sample: hamburgers, meatballs, and minced meat. All the samples were sealed in sterile plastic bags and kept at 4 °C prior to analysis.

The samples (25 g) were aseptically weighed into sterile bags, diluted with 200 mL sterile buffered peptone water, homogenized in a stomacher (Stomacher® 400 Circulator, Seward, London, UK) for about 5 min, and then 0.1 mL was spread onto Slanetz-Bartley agar plates (Liofilchem, Roseto degli Abruzzi, Italy) supplemented with vancomycin (4 μg/mL) and incubated at 37 °C for 24 h. One colony per Slanetz-Bartley agar plate (Liofilchem, Roseto degli Abruzzi, Italy) supplemented with vancomycin with typical enterococcal morphology was identified to the genus level by cultural characteristics, Gram staining, a catalase test, and bile-esculin reaction.

### 2.2. Antimicrobial Susceptibility Test

Antimicrobial susceptibility was tested by the disk diffusion method [26], according to the recommendations of the Clinical and Laboratory Standards Institute [27] for enterococci. The susceptibility of each enterococcal isolate was tested for 11 antibiotics: vancomycin (30 mg), teicoplanin (30 mg), ampicillin (10 mg), streptomycin (300 mg), gentamicin (120 mg), kanamycin (120 mg), chloramphenicol (30 mg), tetracycline (30 mg), erythromycin (15 mg), quinupristin-dalfopristin (15 mg), and ciprofloxacin (5 mg). Only the category of high-level resistance was considered for streptomycin, gentamicin, and kanamycin. *Enterococcus faecalis* strain ATCC 29212 and *Staphylococcus aureus* strain ATCC 25923 were used as quality control organisms.

### 2.3. Whole-Genome Sequencing, Genome Assembly, and Gene Detection

Bacterial DNA was extracted from overnight cultures with a DNeasy Ultraclean Microbial Kit ™ (Qiagen, Hilden, Germany) as recommended by the manufacturer. The whole genome sequence (WGS) of strains was determined by de novo assembly of 2 x 150-bp paired-end reads generated with Illumina sequencing technology (San Diego, CA, USA) using assembler SPAdes [28], Burrows-Wheeler Aligner [29], and Pilon [30] for final polishing. The antibiotic resistance genes were characterized with the ARIBA [31] package by mapping short reads against a manually curated and updated database of resistance-associated genes derived from CARD and Resfinder [32,33].

### 2.4. MALDI-TOF Mass Spectrometry

Bacterial growth was performed on Levine agar medium with and without antibiotic, following the CLSI standards [27]. The protein extracts were obtained from intact bacterial cells using a quick method described by Freiwald and Sauer [34]. The analyses were performed with an Autoflex Speed (Bruker Daltonics, Bremen, Germany) by using the following parameters: linear mode, positive-ion extraction with voltage 19.56 kV at source 1 and 18.09 kV at source 2, delay time about 160 ns. For each extracted colony, a spectrum was obtained from the sum of three thousands laser shots at the frequency of 1000 Hz. A calibrant spot was analyzed before each isolate analysis by summing four thousands laser shots at the frequency of 1000 Hz.

### 2.5. Statistics and Bioinformatics Analysis of Spectra

The sample mass spectra were analyzed using ClinProToolsTM software (version 3.0, Bruker Daltonics, Bremen, Germany), which includes three types of machine learning algorithms to generate classification models: quick classifier (QC), genetic algorithm (GA), and supervised neural network (SNN). The selected spectra were submitted to the three algorithms, QC, GA, and SNN, where the cross validation is based on the precision of the algorithm in the correct assignment of a random sample to the correct group. For each peak, the analysis of the operational characteristic of the receiver (ROC) was performed based on the area under the ROC curve (AUC).

## 3. Results

### 3.1. Bacterial Isolation

A total of 19 vancomycin-resistant enterococci isolates were recovered from the 129 samples of processed meat. The distribution was the following: seven isolates from hamburgers; six isolates from meatballs; and six isolates from minced meat. After mass spectrometry species identification, we identified 14 isolates of *Enterococcus faecium*, three isolates of *Enterococcus gallinarum,* and two isolates of *Enterococcus durans*.

### 3.2. Antibiotic Susceptibility Study

All strains showed resistance to three or more antimicrobials, in addition to vancomycin. Higher incidence of resistance was observed for teicoplanin (n = 17), erythromycin (n = 16), and tetracycline (n = 16). Additionally, fourteen isolates showed resistance to ampicillin, and six were resistant to ciprofloxacin. High-level resistance to aminoglycosides was also detected in two isolates that showed high-level resistance to kanamycin and gentamicin and one isolate that showed high-level resistance to streptomycin. Furthermore, all isolates were susceptible to chloramphenicol and quinupristin-dalfopristin.

### 3.3. Detection of Antibiotic Resistance Genes

After whole-genome sequencing, with Illumina technology (San Diego, CA, USA), and *de novo* assembly, we detected that sixteen of the nineteen VRE isolates (*E. faecium and E. durans*) were *vanA* positive and two *E. gallinarum* were *vanC1* positive. Interestingly, one *E. gallinarum* isolate showed the combination *vanA* + *vanC1* (Table 1).

Regarding the genes that confer resistance to aminoglycoside antibiotics, we identified the presence of the *aac*(6’)-*Ii* gene in all *E. faecium* isolates and in one *E. gallinarum* isolate (79%; n = 15/19), the *ant*(9)-*Ia* gene in six *E. faecium* isolates and in one *E. gallinarum* isolate (37%; n = 7/19), the *aadE* gene in six *E. faecium* isolates (32%; n = 6/19), the *aac*(6’)-*Iih* gene in only the *E. durans* isolates (11%; n = 2/19) and the *aac*(6’)-*Ie*-*aph*(2’’)-*Ia* gene in only one *E. faecium* isolate (5%; n = 1/19) (Table 1).

Several genes that confer resistance to macrolides were detected. We identified in most of the isolates the *erm*(B) gene (89%; n = 17/19). We also detected in one *E. faecium* isolate the *erm*(T) gene (5%; n = 1/19). Other macrolide resistance genes were detected, such as the *lsa*(E) gene in six *E. faecium* isolates (32%; n = 6/19), the *mef*(A) gene in only one *E. gallinarum* isolate (5%; n = 1/19), the *msr*(C) gene in all *E. faecium* isolates (74%; n = 14/19), and the *msr*(D) gene in only one *E. gallinarum* isolate (5%; n = 1/19) (Table 1).

We also detected one licosamide resistance gene, *lnu*(B), in six *E. faecium* isolates (32%; n = 6/19) (Table 1).

A combination of the *tet*(M) + *tet*(L) genes, conferring resistance to tetracycline, was found in all *E. duran* isolates, in nine *E. faecium* isolates, and in one *E. gallinarum* isolate (63%; n = 12/19) (Table 1).

Considering the lipopeptides class and the *E. faecium* species, since they were only detected in this species, the *cls* gene was present in all isolates (74%; n = 14/19), the *liaS* gene in nine isolates (47%; n = 9/19), and the *liaR* gene in only one isolate (5%; n = 1/19) (Table 1).

Some trimethoprim resistance genes were also detected, such as the *dfrG* gene in two *E. faecium* isolates (11%; n = 2/19) and the *dfrK* gene in one *E. faecium* isolate (5%; n = 1/19). We also identified two genes that are involved in efflux pump complexes, such as *adeC* (74%; n = 14/19) and *efmA* (74%; n = 14/19), present in all *E. faecium* isolates (Table 1).

### 3.4. Putative Biomarkers of Resistance Detection

**Figure 1:**
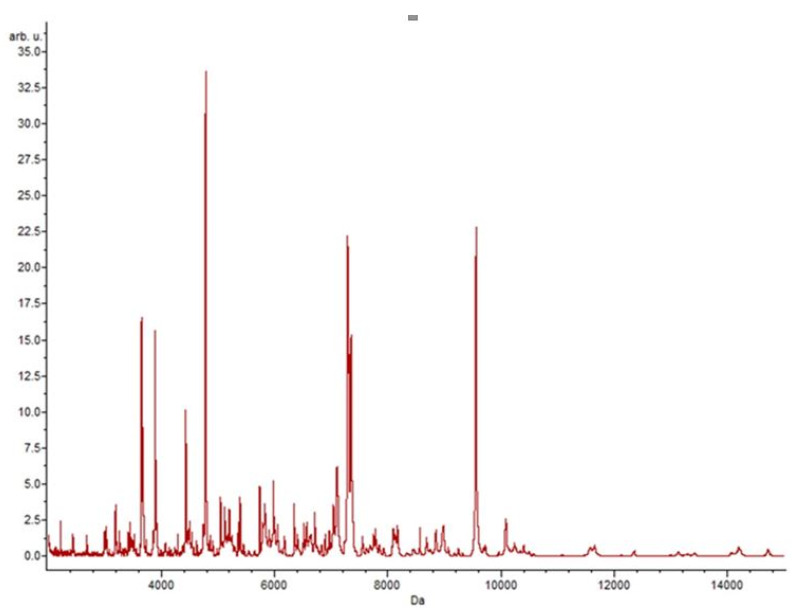
Representative spectrum of *Enterococcus* spp. without antibiotic.

The aim of this approach was to observe the mass profiles of the resistance; therefore, an analysis was made using all isolates that showed resistance to a certain antibiotic. Data analysis was always done by comparing two classes: class 1 represents the isolates that grew in the absence of an antibiotic in the culture medium, and class 2 represents the isolates that grew in the presence of an antibiotic. After submission to the various classification algorithms, it was in the presence of erythromycin that more specific peaks were found (n = 28). In contrast, it was in the presence of ciprofloxacin that a lower number of specific peaks was detected (n = 11). Figure 1 shows the representative spectrum of the enterococci strains without antibiotic.

**Figure 2:**
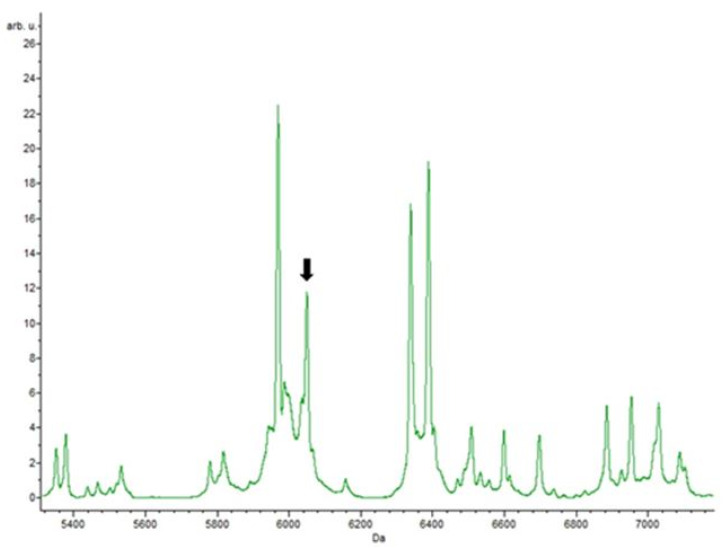
*Enterococcus faecium* spectrum showing the peak m/z 6048 ± 1 (*p*-value is 0.00001) characteristic of these strains.

For all enterococci isolates, with and without antibiotics, a peak at m/z 4424.01 ± 3 was observed, representing a pertinent biomarker of the Enterococcus genus. A peak at m/z 6048 ± 1 was detected in all samples of *E. faecium* isolates, with a *p*-value of 0.00001, making this peak statistically significant and pointing to this as a putative biomarker of this species (Figure 2).

**Figure 3:**
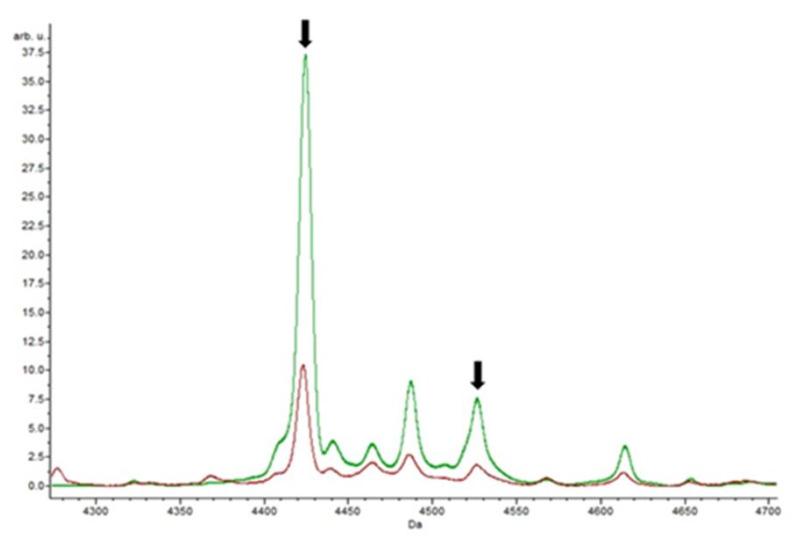
Peaks obtained for tetracycline. From the analysis of the ClinProTools software, the control spectrum without antibiotics (red) and the spectrum with the action of tetracycline (green) were obtained. The results show statistically significant peaks, with a *p*-value of 0.0000001, such as masses m/z 4424.01 and m/z 4526.45 noted by arrows.

The eighteen isolates resistant to tetracycline were analyzed by MALDI-TOF MS, and out of the 66 spectra analyzed, only 12 peaks were detected in at least one classification algorithm as potential biomarkers. Four of these peaks, m/z 2971.34, m/z 4424.01, m/z 4526.45, and m/z 6036.59, were recognized by two of the algorithms simultaneously. Figure 3 is a focus of the spectrum showing the masses m/z 4424.01 and m/z 4526.45.

The peak at m/z 6036.59 was exclusively detected in the isolates resistant to tetracycline. The specificity of this peak, regarding the antibiotic, may indicate a potential biomarker of resistance. In contrast, the peak at m/z 4526.45 ± 1 was also identified in the isolates resistant to erythromycin, and the peak at m/z 2971.34 ± 1 in the isolates resistant to teicoplanin and erythromycin.

Relative to the isolates resistant to erythromycin, the peak that stood out the most, of a total of 165 peaks detected, was at m/z 4423.39, since all classification algorithms detected it and the AUC value was 0.92. Four unique peaks were also identified, m/z 3289.2, 5951.28, m/z 6387.81, and m/z 6926.39, with great intensity relative to the control isolates.

In a model generation of each algorithm, there was a model internally validated by cross-validation. Herewith, the validation values for the model correspond to 97.77% for the genetic algorithm, 90.04% for the supervised neural network and 87.32% for the QuickClassifier, which proves the accuracy of the peaks obtained for erythromycin.

Of the 21 peaks detected in the isolates resistant to teicoplanin, only five of them were considered putative biomarkers as shown in Table S1. Two of them were found in the three classification algorithms, and the other three peaks were only detected by two classification algorithms (Table S1).

**Figure 4:**
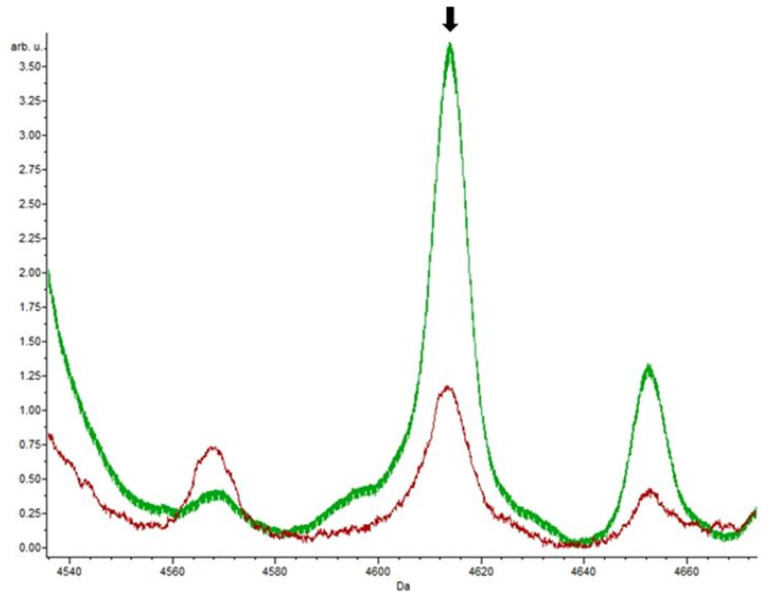
The red spectrum represents the control and the green spectrum was obtained by the action of teicoplanin. The arrow shows a peak at m/z 4652.66 with greater intensity in the presence of the antibiotic.

Furthermore, the peaks at m/z 2153.18, m/z 4227.42, and m/z 4652.66 were not detected simultaneously by the three classification algorithms; however, they can be used as biomarkers of resistance since they were only identified in isolates resistant to this antibiotic. Nevertheless, the values of the AUC curve were not as significant (0.63, 0.71, and 0.54, respectively), which means these peaks can be false positives. Figure 4 focuses on the part of the spectrum highlighting the peak at m/z 4652.66.

None of the peaks detected in the isolates resistant to ciprofloxacin was identified by more than one classification algorithm. However, the cross validation in the model generation was 94.74% for the genetic algorithm, 60.09% for the supervised neural network, and 87.03% for the QuickClassifier, demonstrating that although the peaks were only found by one of them, the peaks obtained were accurately detected. This result may be due to the fact that the total number of analyzed spectra was lower (n = 24) than in isolates resistant to other antibiotics.

**Figure 5:**
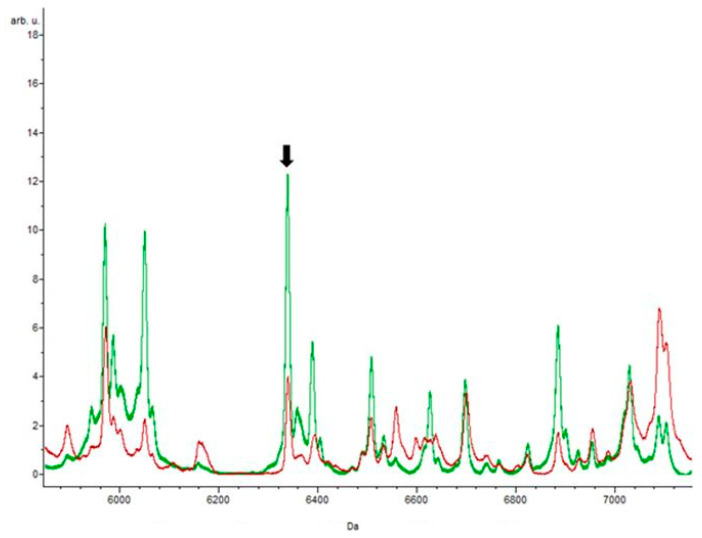
The red spectrum represents the control, and the green spectrum was obtained by the action of ciprofloxacin. The arrow shows the peak at m/z 4652.66 with greater intensity in the presence of the antibiotic. Among the 27 specific peaks detected in the isolates resistant to ampicillin, only the peak with mass m/z 6338 was identified by the three classification algorithms, with an area under the curve (AUC) value of 0.89. This peak was also identified in erythromycin-resistant isolates. In contrast, three other peaks, m/z 2361.38, m/z 3304.92, and m/z 7240.29, proved to be exclusive to these isolates, which means they are potential biomarkers of ampicillin resistance.

The peaks at m/z 6358.27 and m/z 13237.3, with a p-value of 0.0000001, were only present in the isolates resistant to this antibiotic, indicating potential biomarkers. The peak at m/z 6358.27 appeared in isolates stressed with ciprofloxacin, with great intensity, suggesting that it is a protein underlying this resistance mechanism since it was absent in the control (Figure 5). In contrast, in relation to the control, the peak at m/z 13237.3 decreased in intensity. 

**Figure 6:**
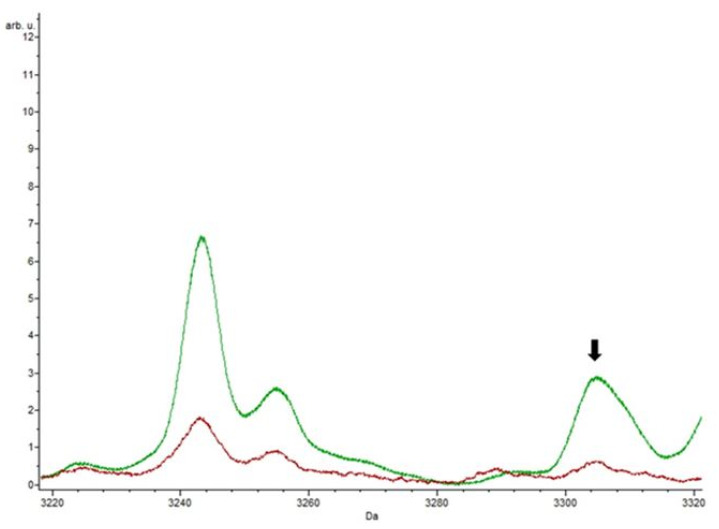
The red spectrum represents the control, and the green spectrum was obtained by the action of ampicillin. The arrow shows the peak at m/z 3304.92 that was only detected in the presence of the antibiotic.

Figure 6 focuses on the part of the spectrum highlighting the peak at m/z 3304.92.

**Figure 7:**
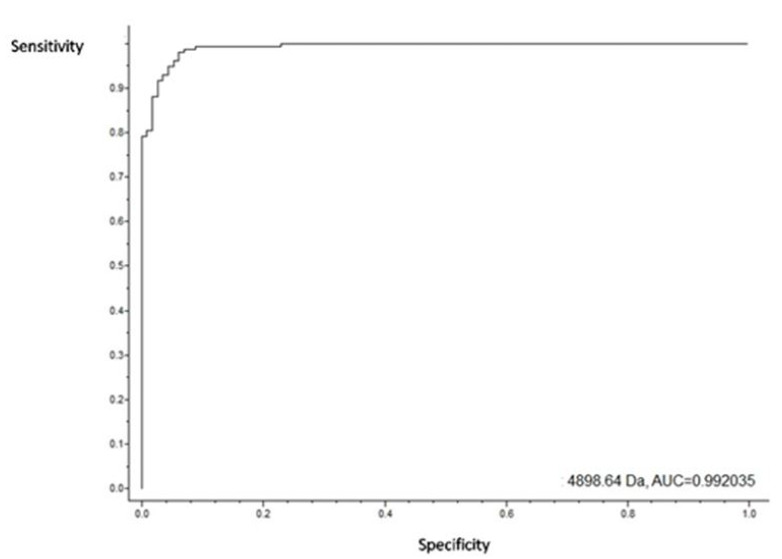
AUC value for the peak at m/z 4898.64. The value of the area under the ROC curve is 0.99, indicating a high-test pass for detecting the peak at m/z 4898.64.

It was among the isolates resistant to vancomycin that the greatest number of peaks was detected, about twenty-five. The peak at m/z 4898.64 was shown to have the highest AUC value (0.99) (Figure 7).

The peak at m/z 4898.64 appeared in all antibiotics except the teicoplanin antibiotic, referring to the absence of the protein in the specific resistance mechanism of this antibiotic. The cross validation in the model generation was 97.61% for the genetic algorithm, 96.65% for the supervised neural network, and 94.48% for the QuickClassifier, proving the efficiency of our model regarding the analysis of the isolates resistant to this antibiotic. It was also found that the peaks at m/z 2263.54 and m/z 7610.17 were only present when these bacteria were stressed with vancomycin. The peak at m/z 2263.54 showed an increase in the intensity relative to the control isolates, which proves that this peak is a potential biomarker of vancomycin resistance.

The masses m/z 3255.03, m/z 3454.55, m/z 3665.36, m/z 5352.64, m/z 5988.57, m/z 8738.67, m/z 9234.06, m/z 9950.35, m/z 10072.31, m/z 10224.25, m/z 12209.66, and m/z 12983.05 were detected in isolates resistant to vancomycin, but also in the control isolates. The peaks at m/z 3255.03, m/z 3665.26, and m/z 5352.64 increased in intensity relative to control isolates, while the remaining peaks decreased in intensity, which may indicate that these peaks are potential vancomycin resistance biomarkers.

## 4. Discussion

Currently, the detection of *Enterococcus* spp. resistant to vancomycin is generally considered an epidemiological problem since they mostly reside in the human gastrointestinal tract and can persevere in hospital environments. Colonization with VRE generally precedes infection [35]. Interest in the detection of VRE has been increasing [36], given the global relevance of the problem. In Portugal, this resistance has been reported in several environments, with special incidence in wild animals from our research group [37,38,39,40].

Interestingly, the species *Enterococcus faecalis* was not identified among the VRE strains. This result is in accordance with other studies carried out in animal-originated food [41,42,43]. In contrast, the presence of *E. faecalis* was reported by researchers in samples of meat in Tunisia [44] and other food products of animal origin in Turkey [45]. The predominant species of enterococci in the processed meat samples used in this study was *E. faecium*. This fact is corroborated by data previously reported for chicken meat, different types of meat, and ready-to-eat seafood [42,46,47].

All VRE isolates presented a phenotype of multi-resistance, where 17 isolates showed resistance to teicoplanin, 16 isolates to erythromycin and tetracycline, and 14 isolates to ampicillin. The high use of these antibiotics, particularly erythromycin, in human and animal medicine, may explain the increasing level of resistance [5,48]. Ciprofloxacin resistance had low incidence, similar to other studies [49]. The fact that fewer isolates showed resistance to ciprofloxacin when compared to the antibiotics mentioned above can be explained by its moderate activity on enterococci [50].

High-level resistance to aminoglycosides was also identified: gentamicin (n = 2), kanamycin (n = 2), and streptomycin (n = 1). Another study also detected VRE isolates that showed high-level resistance to aminoglycosides; however, the resistances were only for kanamycin and streptomycin [48]. Additionally, all isolates from this study, similar to many other clinical isolates from other studies, remained susceptible to chloramphenicol [51,52,53]. This susceptibility can be explained by the availability of specific therapies that make the clinical use of this antibiotic less common [54]. The species *E. faecium* showed phenotypic resistance to most of the antibiotics under study, with 12 isolates resistant to tetracycline, 11 isolates to erythromycin, six isolates to ciprofloxacin, and two isolates with high-level resistance against aminoglycosides. *E. durans* was shown to be resistant to erythromycin, tetracycline, and ampicillin. Although *E. durans* is one of the species causing the lowest percentage of infections [55], this species has been achieving a level of resistance compared to *E. faecium*, which leads to considering this risk, apparently smaller, more concerning. The *E. gallinarum* species showed resistance to antibiotics such as erythromycin, tetracycline, and ampicillin. One of the isolates (H6) also showed a high level of resistance to streptomycin. The presence of vancomycin resistance in *E. gallinarum* was expected since this species was identified by the presence of the *vanC1* gene, which confers a low level of intrinsic resistance to this antibiotic. Similar to *E. durans*, the presence of high levels of antibiotic resistance makes *E. gallinarum* a target of attention.

With respect to the resistance genes obtained from Next-Generation Sequencing (NGS), in this study we detected a wide variety of genes conferring resistance to multiple antimicrobial agents.

Vancomycin and ampicillin resistance is among the most common and problematic resistance in enterococci, especially in *E. faecium*. While the *vanA* and *vanB* genes, associated with vancomycin resistance, are considered clinically relevant in *E. faecium* and *E. faecalis*, the *vanC1* gene is an intrinsic gene of *E. gallinarum* [54]. The detection of *vanA* and *vanC1* genes in this study confirms the presence of VRE among our processed meat isolates, corroborating, in this way, our phenotypic results. Most *vanA*-positive isolates were *E. faecium;* however, this gene was also detected in *E. durans* isolates and, interestingly, in one *E. gallinarum* isolate. Similarly, in a study carried out on raw meat products in Italy, the presence of a *vanA* + *vanC*1 combination in one *E. gallinarum* isolate was also reported [56]. These results suggest that not only *E. faecium*, but also other *vanA*-positive enterococci, are present in meat products and are potentially capable of disseminating to humans.

The most prevalent aminoglycoside resistance gene among our isolates was *aac*(6’)-*Ii* (79%). In a study carried out on enterococci isolated from bird carcasses, the presence of *aac*(6’)-*Ii* was detected in most isolates (32%) [57]. We identified one isolate that harbored the *aac*(6’)-*Ie*-*aph*(2’’)-*Ia* gene and six isolates that harbored the *aadE* gene, as other researchers did in a comparative study between isolates from humans, pigs, and pork where they also detected these two genes in *E. faecium* and in *E. faecalis* [58]. We also detected two more genes that confer resistance to aminoglycoside antibiotics, namely, *aac*(6’)-*Iih* and *ant*(9)-*Ia*. A study carried out in bovine feces also reported the presence of the *aac*(6’)-*Iih* gene in 10 isolates of *E. hirae*, whereas in our study it was found in *E. durans*, and the *ant*(9)-*Ia* gene was found in only one isolate of *E. faecium* in the same way as in our study [59].

Resistance to macrolide antimicrobials was confirmed mostly by the presence of *erm*(B) genes; however, we also detected other genes, namely, *erm*(T), *msr*(C), *lsa*(E), *mef*(A), and *msr*(D). Other studies also reported the presence of these genes among samples isolated from meat in Tunisia [43] and in fecal samples from animals for consumption, such as cattle and pigs [59,60]. Although these studies were not carried out on meat, it is also important to underline that these animals will serve as food for humans and will possibly transmit this resistance.

Other genes were detected in this work, such as *lnu*(B), *msr*(C), *msr*(D), *cls*, *liaS*, *liaR*, *dfrG*, *dfrK*, *efmA,* and *adeC*. Other researchers also detected the presence of some of these genes in enterococci, for example, Cavaco and his collaborators, in 2017, reported the presence of *lnu*(B) and *msr*(C) genes among *E. faecalis* isolates [61]; López along with colleagues, reported in 2012 the presence of *dfrG* and *dfrK* genes in enterococci isolates [62]; Beukers and collaborators, in 2017, detected the presence of the *adeC* gene among *E. faecium* isolates [59]; and *cls*, *liaS,* and *liaR* were detected by Lellek and colleagues in 2015, also among *E. faecium* isolates [63].

The genes found in our NGS analysis agreed with some the resistance phenotypes observed. However, in some isolates, we identified the resistance phenotype but no resistance gene, and in others we verified that in some isolates we detected resistance genes but no resistance phenotypes. Nevertheless, there is not always a correlation between phenotype and genotype. Sometimes, the susceptibility to antibiotics is highly dependent on the bacterial metabolism, and the global metabolic regulators that modulate this phenotype or resistance genes may not be expressed [64].

These results indicate that consumers are exposed to VREs from various animal-origin foods. This can be directly through the consumption of contaminated food or indirectly through cross-contamination with other foods during processing.

Considering the detection of resistance biomarkers, for the aminoglycoside antibiotics class, it was not possible to carry out the MALDI-TOF MS analysis, since the number of isolates resistant to this antibiotics class did not indicate a statistically significant result.

In this work there were masses detected by the different classification algorithms that were present in different antibiotics. Possibly, these masses corresponded to basal proteins of the bacteria, since they were present in isolates resistant to different antibiotic classes [25]. All peaks detected for the genus (m/z 4424.01 ± 3) and *E. faecium* (m/z 6048 ± 1) were in accordance with those reported in the literature, representing a good biomarker [65,66].

In a previous study on MRSA, it was observed that masses below m/z 2400 were more intense in MRSA susceptible to teicoplanin compared to MRSA resistant to teicoplanin [67]. The results obtained in this work are in accordance with this study about teicoplanin, since only two peaks with a mass below this value were detected in our teicoplanin-resistant isolates.

A study carried out in 2012 by Griffin, aiming to determine the difference between the masses of *vanA* and *vanB* genes. A peak at m/z 6603.51 was observed more intensely in *vanA* compared to *vanB* [68]. In our study on vancomycin, as the *vanB* gene was not present, we were unable to make this distinction.

Many of the peaks obtained, although not exclusive, are considered good putative biomarkers because there are significant variations, either due to the increase or decrease in the intensity of the peaks, relative to control isolates. For the remaining peaks detected in the isolates resistant to the different antibiotics, there are not many studies that helped us to prove that the putative biomarkers in this work are indicators of resistance biomarkers, which makes this work a good starting point for other researchers. Biomarkers would be of utmost importance to detect antibiotic-resistant bacteria from any reservoir, which could make the use of biomarkers promising in the context of studies on One Health.

## 5. Conclusions

This study confirmed the existence of vancomycin-resistant enterococci strains of different species in processed meat samples. Several studies have reported the reality of this problem in animal production; however, this study confirms the presence and dissemination of these microorganisms in human food. The isolates under study showed multi-resistance to antibiotics in addition to being vancomycin resistant. 

The results obtained from advanced techniques, such as next-generation sequencing, suggest that meat plays a potential role as a reservoir of resistance genes, triggering the need to carry out more studies to evaluate the mobility of these genes. MALDI-TOF MS demonstrated high potential for the identification of putative biomarkers of resistance to different antibiotics. Thus, the consumption of contaminated meat may be associated with the spread and colonization of vancomycin-resistant enterococci in humans and for this reason, could represent a public health concern.

## Figures and Tables

**Table 1:** Characteristics of the Nineteen Vancomycin-Resistant Enterococci Isolates Recovered from Processed Meat.

Isolate	Species	Vancomycin Resistance Gene Detected	Resistance	
			Phenotype	Genotype
H1	*Enterococcus faecium*	*vanA*	TET TEI VAN CIP AMP ERY	*aac*(6’)*-Ii*, *tet*(L), *tet*(M), *adeC*, *efmA*, *msr*(C), *cls*, *erm*(T), *erm*(B), *dfrG*
H2	*E. faecium*	*vanA*	TET TEI VAN AMP	*aac*(6’)*-Ii*, *aadE*, *ant*(9)*-Ia*, *tet*(L), *tet*(M), *adeC*, *efmA, lnu*(B), *msr*(C), *cls*, *liaS*, *lsa*(E), *erm*(B)
H3	*E. faecium*	*vanA*	TET TEI VAN AMP	*aac*(6’)*-Ii*, *aadE*, *ant*(9)*-Ia*, *tet*(L), *tet*(M), *adeC*, *efmA*, *lnu*(B), *msr*(C), *cls*, *liaS*, *lsa*(E), *erm*(B)
H4	*E. faecium*	*vanA*	TET TEI VAN CIP AMP	*aac*(6’)*-Ii*, *aac*(6’)*-Ie-aph*(2’’)*-Ia*, *adeC*, *efmA*, *msr*(C), *cls*, *liaS*, *erm*(B), *dfrG, dfrK*
H6	*Enterococcus gallinarum*	*vanA*, *vanC1*	TET TEI VAN AMP ERY STR	*aac*(6’)*-Ii*, *erm*(B)
H7	*E. faecium*	*vanA*	TET VAN AMP ERY CN K	*aac*(6’)*-Ii*, *tet*(L), *tet*(M), *adeC*, *efmA*, *msr*(C), *cls*, *liaS*, *liaR*, *erm*(B)
H8	*E. faecium*	*vanA*	TET TEI VAN CIP AMP ERY	*aac*(6’)*-Ii*, *adeC*, *efmA*, *msr*(C), *cls*, *erm*(B)
A10	*E. faecium*	*vanA*	TET TEI VAN AMP ERY	*aac*(6’)*-Ii*, *aadE*, *ant*(9)*-Ia*, *tet*(L), *tet*(M), *adeC*, *efmA*, *lnu*(B), *msr*(C), *cls*, *liaS*, *lsa*(E), *erm*(B)
A11	*Enterococcus. durans*	*vanA*	TET TEI VAN AMP ERY	*aac*(6’)-*Iih*, *tet*(L), *tet*(M), *erm*(B)
A12	*E. gallinarum*	*vanC1*	TEI VAN AMP ERY	*ant*(9)-*Ia*, *msr*(D), *mef*(A)
A13	*E. faecium*	*vanA*	VAN CIP AMP ERY	*aac*(6’)*-Ii*, *adeC*, *efmA*, *msr*(C), *cls*, *erm*(B)
A14	*E. faecium*	*vanA*	TET TEI VAN ERY	*aac*(6’)*-Ii*, *aadE*, *ant*(9)*-Ia*, *tet*(L), *tet*(M), *adeC*, *efmA*, *lnu*(B), *msr*(C), *cls*, *liaS*, *lsa*(E), *erm*(B)
A16	*E. faecium*	*vanA*	TET TEI VAN CIP AMP ERY CN K	*aac*(6’)*-Ii*, *adeC*, *efmA*, *msr*(C), *cls*, *erm*(B)
CP17	*E. faecium*	*vanA*	TET TEI VAN AMP ERY	*aac*(6’)*-Ii*, *aadE*, *ant*(9)*-Ia*, *tet*(L), *tet*(M), *adeC*, *efmA*, *lnu*(B), *msr*(C), *cls*, *liaS*, *lsa*(E), *erm*(B)
CP18	*E. faecium*	*vanA*	TET TEI VAN ERY	*aac*(6’)*-Ii*, *tet*(L), *tet*(M), *adeC*, *efmA*, *msr*(C), *cls*, *liaS*, *erm*(B)
CP19	*E. faecium*	*vanA*	TEI VAN CIP AMP ERY	*aac*(6’)*-Ii*, *adeC*, *efmA*, *msr*(C), *cls*, *erm*(B)
CP21	*E. faecium*	*vanA*	TET TEI VAN ERY	*aac*(6’)-*Ii*, *aadE*, *ant*(9)-*Ia*, *tet*(L), *tet*(M), *adeC*, *efmA*, *lnu*(B), *msr*(C), *cls*, *liaS*, *lsa*(E), *erm*(B)
CP22	*E. durans*	*vanA*	TET TEI VAN ERY	*aac*(6’)-*Iih*, *tet*(L), *tet*(M), *erm*(B)
CP23	*E. gallinarum*	*vanC1*	TET TEI VAN ERY	*tet*(L), *tet*(M)

TET: tetracycline; TEI: teicoplanin; VAN: vancomycin; CIP: ciprofloxacin; AMP: ampicillin; ERY: erythromycin, STR: streptomycin; CN: gentamicin; K: kanamycin.

## Supplementary Materials

The following are available online at https://www.mdpi.com/2079-7737/9/5/89/s1, Table S1: Exclusive masses found for each antibiotic.
