# Supplementary material for: Next-Generation Sequencing and MALDI Mass Spectrometry in the Study of Multiresistant Processed Meat Vancomycin-Resistant Enterococci (VRE)
Source: Biology (Basel). 2020 Apr 27;9(5):89. doi: 10.3390/biology9050089 (PMC7284646; doi:10.3390/biology9050089)
Supplement: Supplementary file 1 [file biology-09-00089-s001.pdf]

Table S1: Exclusive masses found for each antibiotic

Peak (m/z)	Model			Standard deviation		AUC
	GA	SNN	QC	Class standard deviation 1	Class standard deviation 2	
**Tetracycline**						
6036.59	1.41		8.21E-19	1.42	2.7	0.90
**Teicoplanin**						
2970.87		0.10	1.94E-26	2.48	3.79	0.95
4423.75	1.13	0.01	4.33E-23	21.53	17.95	0.91
4526.36	1.46	0.03	9.98E-25	2.19	3.23	0.94
4652.66	0.12	0.03		0.81	0.92	0.54
6049.18	1.22	0.01		3.23	3.35	0.91
**Ciprofloxacin**						
6358.27	0.49			1.71	1.46	0.77
13237.3		0.02		0.34	0.06	0.68
**Ampicillin**						
2361.38		0.02		1.54	0.61	0.80
3304.92	0.81			1.53	1.27	0.84
7240.29		0.01		1.68	3.14	0.67
**Vancomycin**						
3255.03		0.11		2.14	1	0.73
3454.55	0.98	0.14		1.49	0.78	0.86
3665.36		0.03		12.02	9.91	0.63
5352.64		0.01		8.25	13.6	0.83
5988.57		0.02		2.99	5.1	0.81
8738.67		0.01		1.14	0.04	0.97
9234.06		0.01		1.13	0.25	0.82
9950.35		0.03		0.72	0.07	0.87

10072.31		0.01		8.42	2.06	0.72
10224.25		0.01		1.59	0.53	0.72
12209.66		0.03		0.2	0.03	0.97
12983.05		0.04		0.23	0.01	0.96
